# Deafness and Discharges from the Ear

**Published:** 1892-06

**Authors:** 


Deafness and Discharges from the Ear. The Modern Treatment for the Radical Cure of Deafness, Otorrhea, Noises in the Head, Vertigo, and Distress in the Ear. By Samuel Sexton, M. D., Author of The Ear and its Diseases, etc., assisted by Alexander Duam, M. D. Small octavo, pp. 89. New York: J. H. Vail & Co. 1890.

In this handsome little volume of eighty-nine pages, the author has compressed much information on a subject of great importance to the general practitioner. Even the busy physician must always be prepared to give his patient intelligent advice upon the first symptoms that relate to deafness, and be able to treat the initial stages of the malady with skill and intelligence. Here will be found some of the best thoughts on this important subject, and they can easily be availed of by the masses, for the work is simple and reasonable in price.

